# Does one-on-one interaction with faculty members improve resident communication skills in comparison to virtual self-learning?

**DOI:** 10.51894/001c.144195

**Published:** 2025-09-30

**Authors:** Morgan Steele

**Affiliations:** 1 College of Osteopathic Medicine Michigan State University

## 53

### BACKGROUND

Effective disclosure of bad news and medical errors is a required skill for patient-centered care. The SPIKES protocol was developed to assist physicians in this task (1). There is limited data evaluating methods for teaching residents this essential skill (2, 3).

### OBJECTIVES

We compared resident physicians’ understanding of SPIKES when self-learning via virtual methods to analyzing their implementation of the protocol with faculty members (FM). We also evaluated if better understanding of SPIKES improves patient satisfaction.

### METHODS

Sixty-five Internal Medicine and Transitional Year PGY1 residents were asked to study the SPIKES protocol and practice disclosing bad news prior to completing two simulations. Residents were then scheduled for Case 1, a 15-minute, video-recorded standardized patient (SP) encounter where they employed SPIKES. Residents then self-evaluated their performance and understanding of SPIKES using a 22-item checklist based on components of the SPIKES protocol. A FM reviewed the recording with the resident and discussed performance on each SPIKES component. Residents then re-evaluated their performance using the same checklist. Next, residents completed Case 2 with a second SP. FMs and SPs evaluated residents’ performance in Case 1 and Case 2 using the same SPIKES checklist. The residents’ scores pre and post discussion with the FM, and the SP and FM’s scores from Cases 1 and 2 werecompared.

### RESULTS

Residents’ understanding of SPIKES significantly improved after interacting with and receiving feedback from FMs compared to self-learning virtually, evidenced by an improvement in scores from Case 1 to Case 2 on SP and FM evaluations.

### DISCUSSION

In-person learning is required for mastery of interpersonal skills when compared to virtual learning in PGY1 residents.

